# Mapping Caregiver Needs’ Assessment Tools for Family and Friend Caregivers: A Rapid Scoping Review

**DOI:** 10.3390/ijerph23030300

**Published:** 2026-02-28

**Authors:** Xiaoxu Ding, Rose Alavi Toussi, Fernanda L. F. Dal Pizzol, Angie Grewal, Ashley Hyde, Jasneet Parmar, Sharon Anderson, Puneeta Tandon

**Affiliations:** 1 Department of Medicine, Faculty of Medicine & Dentistry, University of Alberta, Edmonton, AB T6G 2R7, Canada; xiaoxu8@ualberta.ca (X.D.); ralavito@ualberta.ca (R.A.T.); ptandon@ualberta.ca (P.T.); 2 Faculty of Nursing, University of Alberta, Edmonton, AB T6G 1C9, Canada; fenglerd@ualberta.ca (F.L.F.D.P.); asangha@ualberta.ca (A.G.); ac20@ualberta.ca (A.H.); 3 Department of Family Medicine, Faculty of Medicine & Dentistry, University of Alberta, Edmonton, AB T6G 2T4, Canada; jasneet.parmar@albertahealthservices.ca

**Keywords:** family caregivers, caregiver-centered care, co-design, integrated care, implementation strategy, policy framework

## Abstract

**Highlights:**

**Public health relevance—How does this work relate to a public health issue?**

- Family and friend caregiving is a widespread public health issue affecting population wellbeing, service access, and health system capacity.
- When caregivers are not routinely identified and their support needs are not assessed, unmet needs can contribute to caregiver health decline, reduced participation in work/social roles, and avoidable care crises and system use.

**Public health significance—Why is this work of significance to public health?**

- This review shows that many “needs assessment” tools do not consistently measure caregiver-defined support needs and are rarely designed for workflow integration, longitudinal reassessment, or Electronic Medical Record documentation.
- These gaps limit equitable, routine caregiver support across settings and transitions, and the findings clarify what tool features are needed to operationalize caregiver-centered care at scale.

**Public health implications—What are the key implications or messages for practitioners, policy makers and/or researchers in public health?**

- Practitioners and policy makers should use and embed tools that explicitly ask caregivers what support they need and ensure results trigger follow-up actions (referral/navigation), supported by workflow and EMR documentation.
- Researchers should develop and test tools for implementation outcomes (workflow fit, responsiveness over time, equity impacts, and whether assessment leads to supports).

**Abstract:**

Background: Family and friend caregivers provide essential support across health and social care systems but remain inconsistently identified, assessed, and supported in routine practice. Although numerous caregiver needs’ assessment instruments exist, many focus on burden, distress, or preparedness rather than explicitly eliciting caregiver-defined support needs, limiting their utility for care planning, care transitions, and system integration. Methods: We conducted a rapid scoping review to identify and characterize caregiver needs’ assessment tools developed for family and friend caregivers. Searches were conducted in MEDLINE, PsycINFO, CINAHL, Web of Science, Health and Psychosocial Instruments, and the Cochrane Library. Eligible studies described the development, validation, or implementation of instruments designed to assess caregiver needs. Data were extracted on tool characteristics, domains assessed, administration methods, and implementation-relevant features. Item-level content analysis distinguished caregiver-defined support needs from related constructs, including burden, strain, preparedness, and care-recipient monitoring. Results: Forty-three studies describing caregiver needs’ assessment instruments were included (19 instruments; 17 instrument families). Tools varied widely in length, administration, and conceptual framing. Seven domains of caregiver-defined support needs were identified: caregiver health and self-care; emotional and psychological support; information, communication, and navigation; practical and instrumental support; social and relational support; autonomy and life participation; and spiritual, cultural, and existential support. Information and navigation needs were most frequently assessed, while autonomy and spiritual domains were least consistently represented. Many instruments demonstrated construct drift, assessing stressors or impacts rather than explicitly eliciting caregiver-defined support needs. Few tools were designed for longitudinal reassessment, workflow integration, or documentation within electronic medical records. Conclusions: Existing caregiver needs’ assessment tools inadequately support routine, system-integrated caregiver-centered care. Advancing caregiver-centered practice requires tools that explicitly elicit caregiver-defined support needs and are designed for workflow integration, longitudinal use, and interdisciplinary care pathways.

## 1. Introduction

Family and friend caregivers are a foundational yet insufficiently integrated component of health and social care systems [1,2]. Across health, social, and community care settings, including primary care, home care, acute care, transitional care and discharge planning contexts (e.g., short-stay rehabilitation or transitional care units), and other community-based services, family and friend caregivers provide the majority of ongoing support for individuals living with physical or mental illness, disability, or age-related needs. Even in assisted or supportive living, long-term care/continuing care (e.g., nursing homes/assisted living), and hospice/palliative care family caregivers assist with 15 to 40% of the care [3,4]. Their contributions extend beyond assistance with daily activities and emotional support to include medication management, advocacy, care coordination, and system navigation across providers and settings [5,6,7,8,9]. This caregiving labour—primarily unpaid—generates substantial economic value; replacing care provided by family and friend caregivers would cost the federal government an estimated $97.1 billion annually [10,11,12].

Despite their central role in sustaining care delivery, caregivers remain inconsistently identified, assessed, and supported within routine care processes, limiting their recognition as partners in care and constraining health system performance [10,11,12]. Family caregivers frequently experience unmet needs across practical, informational, emotional, social, and financial domains, particularly as care becomes more complex and extends over time [13,14,15,16]. When these needs are not systematically addressed, caregivers experience cumulative strain that negatively affects their physical, mental, and social wellbeing, with downstream consequences for care continuity and health system performance [17,18,19].

Routine identification of caregivers and systematic assessment of their support needs function as public health and health system infrastructure, enabling early detection of unmet needs, prevention of avoidable caregiver health decline, and more reliable continuity of care across settings and transitions. As an integrated care function, caregiver needs’ assessment provides the infrastructure to translate caregiver recognition into actionable supports, referral, and follow-up across the care continuum.

Emerging evidence indicates that caregiver stress and workload, rather than patient acuity alone, are key determinants of outcomes such as successful hospital discharge and safe transitions across care settings [20,21]. At the same time, caregivers’ expanding roles as de facto care coordinators and system navigators have been associated with poorer work–life balance and increased cumulative burden, reflecting the transfer of system integration responsibilities to family caregivers without corresponding structural supports [5,22].

In response to growing recognition of caregivers’ contributions, a wide range of caregiver assessment tools has been developed [23,24,25]. However, much of the literature focuses on burden, anxiety, distress, or quality of life measures, including widely used instruments such as the Zarit Burden Interview and related scales [24,26,27]. While these tools have been instrumental in documenting the impact of caregiving, they are limited in their ability to identify caregivers’ specific, actionable needs or to inform care planning and targeted interventions [28]. Burden-focused approaches also risk framing caregiving primarily through a deficit lens, rather than recognizing caregiving as a skilled, dynamic role that requires proactive and ongoing support.

Person-centered caregiver needs’ assessment tools differ fundamentally from burden-based measures by explicitly identifying gaps between caregivers’ circumstances and available supports, thereby informing care planning, referral, and follow-up [29,30,31]. Such tools are particularly relevant for longitudinal use across primary care, home care, acute care, palliative care, and discharge planning contexts, where caregivers’ roles and needs evolve over time. However, despite longstanding calls to recognize caregivers as partners in care, caregiver needs are rarely assessed routinely, reassessed longitudinally, or documented in ways that integrate with electronic medical records (EMRs) or interdisciplinary workflows [20,32,33]. Even tools frequently cited as best practice, such as the Carer Support Needs Assessment Tool (CSNAT), face persistent challenges in achieving routine, system-level implementation [34]. Studies consistently describe barriers related to workflow disruption, documentation burden, and the marginalization of caregiver assessment within patient-centered clinical systems [35,36]. Care remains overtly patient-centered despite rhetoric of family inclusion [34,37]. As a result, caregiver needs’ assessment remains episodic and poorly embedded in care delivery, limiting its capacity to inform real-time decision-making and continuity of care.

Previous reviews of caregiver needs’ assessment tools have generated important insights but have largely approached caregiver assessment as a measurement or psychometric issue rather than as a function embedded within care delivery [38,39,40,41]. Most reviews have focused on specific conditions (e.g., cancer, dementia, non-communicable diseases), restricted inclusion to self-administered instruments, or applied narrow psychometric criteria. While these reviews consistently note that many instruments conflate caregiver needs with burden or distress and are poorly aligned with clinical practice [28], they have not systematically examined whether caregiver needs’ assessment tools fit within routine clinical workflows, support longitudinal use across care transitions [27,42], or are positioned for integration within interdisciplinary documentation systems or electronic medical records (EMRs). Across existing reviews, evaluation of workflow fit, longitudinal reassessment, and documentation integration is largely absent.

A further limitation of the caregiver assessment literature is conceptual misalignment in how ‘need’ is operationalized. Many tools labeled as needs’ assessments rely on adjacent constructs, such as burden, strain, preparedness, or care-recipient monitoring, as proxies for caregiver support needs. In this review, we therefore distinguish caregiver-defined support needs (i.e., explicit requests for support) from these related constructs to clarify what existing instruments actually measure and what they can support in practice.

To address this gap, this rapid scoping review synthesizes the literature on caregiver needs’ assessment tools for family and friend caregivers, with a specific focus on person-centered instruments that explicitly elicit caregiver-defined support needs rather than proxy indicators such as burden or strain. The objectives are to: (1) identify and describe caregiver needs’ assessment tools developed for family and friend caregivers; (2) map the domains of caregiver-defined support needs assessed by these tools; and (3) examine the extent to which instruments are designed to align with clinical workflows, support longitudinal assessment across care transitions, and enable documentation within EMRs across primary care, home care, acute care, palliative care, and discharge planning contexts. A rapid scoping review methodology was selected to efficiently map the scope and characteristics of existing tools, identify implementation-relevant gaps, and generate timely, practice- and policy-relevant insights to support the integration of systematic caregiver needs’ assessment within care delivery.

## 2. Materials and Methods

### 2.1. Study Design

This review employed a rapid review approach, defined as a form of knowledge synthesis in which selected components of the systematic review process are streamlined to produce evidence in a shortened timeframe [43,44,45,46,47]. Rapid scoping reviews are particularly appropriate when the objective is to map and characterize existing evidence, rather than to evaluate intervention effectiveness or conduct meta-analysis [47,48,49,50].

In this study, the rapid scoping review approach was selected to support a timely and structured synthesis of existing caregiver needs’ assessment instruments, with a focus on their purpose, conceptual framing, and key design characteristics. The aim was to generate practice- and policy-relevant insights to inform future tool selection, development, and implementation across health, community, and social care settings. Methodological choices prioritized breadth and relevance of instrument identification over exhaustive retrieval of all possible sources [47].

This scoping review was not formally registered, as registration is not routinely required for scoping or other ‘Big Picture’ reviews, particularly in rapid contexts; however, methodological transparency was ensured through a pre-specified protocol and detailed reporting of any methodological refinements during the review process [47].

### 2.2. Search Strategy

A comprehensive search strategy was developed in collaboration with an experienced research librarian. Electronic searches were conducted on 20 June 2025 in the following databases: MEDLINE (Ovid), PsycINFO, CINAHL, Web of Science, Health and Psychosocial Instruments (HAPI), and the Cochrane Library. Search concepts combined terms related to caregiver needs, unpaid caregiving roles, and measurement instruments. Caregiver-related terms included caregiver, family caregiver, informal caregiver, unpaid caregiver, care partner, care partner, and carer. Measurement-related terms included scale, instrument, checklist, questionnaire, tool, measure, and assessment.

To enhance comprehensiveness and address indexing variability, database searches were supplemented with backward and forward citation searching, conducted independently by at least two reviewers. Full search strategies for all databases are provided in Supplementary Materials: File S1. Search Strategy.

### 2.3. Eligibility Criteria

Eligibility criteria were structured using the Population–Concept–Context (PCC) framework. The population included family caregivers, such as family members, chosen family, friends, or neighbours who provide unpaid care or support to individuals with health-related needs. The concept focused on instruments designed to systematically assess caregiver needs, broadly defined, rather than tools limited to caregiver burden, stress, quality of life, or disease-specific self-management. The context included any health, community, or social care setting.

Eligible studies comprised empirical research describing the development, validation, implementation, or evaluation of caregiver needs’ assessment instruments, using qualitative, quantitative, or mixed methods designs. Only peer-reviewed articles published in English were included. Grey literature was excluded due to feasibility constraints inherent to the rapid scoping review methodology.

Studies were excluded if instruments:

(a) Measured constructs other than caregiver needs (e.g., burden, preparedness, quality of life);
(b) Focused primarily on disease-specific self-management tasks (e.g., diabetes care);
(c) Targeted paid or professional caregivers.

Although studies were excluded if instruments were explicitly designed only to measure constructs such as burden, strain, or preparedness, we included instruments that were presented, labeled, or positioned by authors as caregiver “needs” assessments (or used as such in practice). We then used item-level content analysis to differentiate caregiver-defined support needs from proxy constructs (e.g., stressor screening, preparedness/capability, care-recipient monitoring), which we report as patterns of construct drift. Conference abstracts, editorials, and study protocols without reported results were also excluded.

### 2.4. Screening

All records were imported into Covidence for deduplication, screening, and data management. A pilot screening of 400 records (10%) was conducted to refine eligibility criteria. Two reviewers then independently screened an additional 400 records (10%) to ensure calibration, achieving 98% agreement. Following calibration, remaining titles and abstracts were screened by a single reviewer, while all full-text articles were independently reviewed by two reviewers.

**Figure 1:**
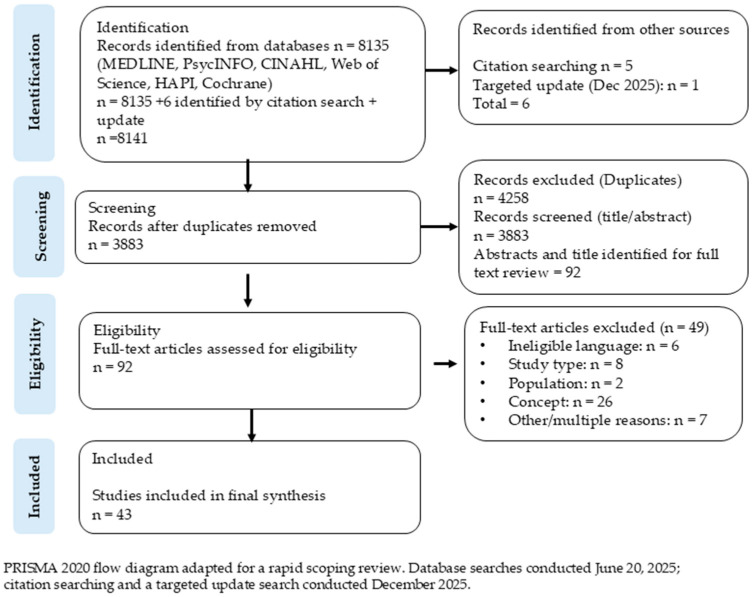
PRISMA 2020 flow diagram (modified for rapid scoping review) showing study identification, screening, eligibility, and inclusion.

Reasons for exclusion at the full-text stage were documented. Disagreements were resolved through discussion and, when necessary, consultation with the broader research team. The study selection process is summarized using a PRISMA flow diagram (Figure 1).

### 2.5. Data Extraction

Two reviewers independently extracted data using Covidence. Extracted variables included: instrument name and purpose; domains assessed; author(s); country and year of development; study population and setting; administration method (e.g., self-administered, clinician-led); number of items; time to administer; mode of delivery; tool language; psychometric properties; and training requirements. Item-level content from each instrument was also extracted to support domain mapping and construct analysis. Discrepancies were resolved through discussion.

### 2.6. Data Analysis and Synthesis

Extracted data were organized into summary tables to capture instrument characteristics and implementation features. A thematic mapping approach was used to identify domains of caregiver need across instruments. Item-level content was examined collectively, and conceptually similar items were grouped based on their underlying focus.

The seven caregiver support need domains were developed as analytic (descriptive) categories to synthesize item-level content across instruments rather than to propose a normative taxonomy of “what caregivers should need.” Domain development was primarily inductive, grounded in iterative coding and grouping of conceptually similar support need items across instruments, and was informed by prior caregiver needs and supportive care literatures to support interpretability and comparison. Domains were retained as separate categories when item content reflected distinct support needs (e.g., autonomy/life participation; spiritual/cultural/existential) that are often collapsed in broader psychosocial groupings. See Supplementary Files for domains, explanations, and examples: File S2. Categorized domains of existing caregiver need items, explanation, and illustrative examples.

To preserve analytic clarity, a distinction was maintained between items that explicitly elicited caregiver needs for support and items reflecting related but distinct constructs (e.g., caregiving capability, care-recipient monitoring, stressor screening, or wellbeing impacts). Instruments were coded as covering a domain only if they included at least one item framed as a caregiver support need. Frequencies and proportions were calculated to summarize domain coverage across instruments. Implementation characteristics and construct drift patterns were synthesized narratively.

To enhance conceptual clarity, caregiver support needs related to autonomy, identity, and life participation and to spiritual, cultural, and existential concerns were retained as distinct domains rather than collapsed into broader psychosocial categories, consistent with item-level coding and prior conceptual work distinguishing support needs from wellbeing impacts or caregiving strain.

## 3. Results

### 3.1. Study Selection

A total of 8141 records were identified, including 8135 records retrieved from electronic database searches and six records identified through other methods (five through citation searching and one through a targeted update search conducted in December 2025). After removal of duplicates (*n* = 4258), 3883 titles and abstracts were screened. Of these, 3797 records were excluded at the title and abstract stage. A total of 92 full-text articles were assessed for eligibility. Of these, 49 articles were excluded for the following reasons: ineligible language (6), study design (8), population (2), concept (26), or other multiple reasons (7). This resulted in 43 articles being included in the final review. The updated PRISMA diagram reflects the inclusion of six additional studies identified through citation searching and the December 2025 update search, now fully accounted for in the totals (Figure 1).

### 3.2. Study Characteristics

Included studies were published between 1995 and 2025 and represented 21 countries/regions, including Australia, Austria, Canada, China, Colombia, Denmark, Germany, Hong Kong, Ireland, Italy, Japan, Malaysia, Mexico, the Netherlands, Norway, Spain, Sweden, Switzerland, Taiwan, the United Kingdom, and the United States.

Most studies used English-language instruments. Non-English tools included Mandarin Chinese (n = 6), Cantonese Chinese (n = 1), Japanese (n = 1), Spanish (n = 3), French (n = 1), Malaysian (n = 1), Dutch (n = 2), German (n = 3), Italian (n = 1), Norwegian (n = 1), and Swedish (n = 1), corresponding to 20 studies overall.

Sample sizes ranged from 11 to 1301 participants. Most instruments targeted caregivers of adults with chronic or life-limiting conditions, while a smaller subset focused on caregivers of children with disabilities (n = 3, 8.1%). Studies were conducted across diverse settings, including home-based care, community organizations, hospitals, hospice and palliative care services, and non-governmental organizations. Reporting of setting varied widely and was not mutually exclusive.

Across the 43 studies, research aims included instrument development and validation (n = 23, 53.5%), cross-cultural adaptation (n = 5, 11.6%), and implementation or feasibility evaluation (n = 15, 34.9%). Thirty-one studies (72.1%) reported at least one psychometric property, most commonly internal consistency (Cronbach’s α).

### 3.3. Overview of Identified Instruments

We identified 19 unique caregiver needs’ assessment instruments across the 43 included studies (Table 1). For analytic purposes, we distinguished between 17 unique instrument families and 20 instrument entries used for domain mapping, treating adaptations or context-specific versions as separate entries when item content differed (Table 2); for example, CHAT and CHAT-D were mapped as distinct entries. Instruments varied substantially in length, domain structure, and administration method, ranging from brief screening tools (7–20 items) to comprehensive assessments exceeding 70 items. While all instruments addressed multiple dimensions of caregiver need, their conceptual orientation varied. Some tools explicitly elicited caregiver-defined support needs (e.g., CSNAT), while others emphasized screening for stressors (e.g., CNST-11) or caregiving preparedness (e.g., MYLOH).

Collectively, Table 1 shows substantial variation in length and administration approach, with relatively few instruments explicitly designed for brief use, longitudinal reassessment, or routine documentation. Administration approaches included self-administration, clinician-guided interviews, and facilitated conversations. Ten studies reported formal training requirements for tool use, particularly in clinical or community service contexts. Reported administration time ranged from 3 to 90 min, with substantial variability both between and within instruments.

### 3.4. Mapping of Caregiver Needs Domains

These domains represent an item-derived analytic synthesis used to map instrument coverage, not a prescriptive framework. For instrument-level analyses, we distinguished between unique instrument families (*n* = 17) and instrument entries used for domain mapping (*n* = 20). Distinct adaptations or context-specific versions of the same instrument were treated as separate entries when they contained non-overlapping or meaningfully revised item content. Unique instrument families are summarized in Table 1, while Table 2 reflects the 20 instrument entries used for domain mapping.

Seven caregiver support need domains were identified following item-level synthesis. These domains reflect conceptual distinctions in the caregiver needs literature and were retained to preserve analytic clarity rather than collapsed into broader psychosocial categories. Domain coverage varied substantially across instrument entries. See Supplementary File S3 for item-level mapping to conceptual domains File S3. Item-level mapping of included caregiver needs assessment instruments to conceptual domains.

The most frequently represented domain was information, communication, and navigation support, appearing in 11 instrument entries (55%), reflecting caregivers’ needs for illness-related information, guidance for decision-making, and clarity about available services and emergency contacts. Practical and instrumental support needs were identified in 10 entries (50%), including assistance with finances, legal matters, employment, transportation, and household tasks. Emotional and psychological support needs were addressed in 9 entries (45%), capturing caregivers’ needs for support in managing distress, worry, and uncertainty.

Caregiver health and self-care and social and relational support were each represented in 8 entries (40%), highlighting moderate attention to caregivers’ own physical wellbeing and their need for support in navigating family relationships and social connections. Autonomy, identity, and life participation needs were less consistently assessed, appearing in 7 entries (35%). Spiritual, cultural, and existential support needs were also among the least frequently represented domains (7 entries; 35%) and were often limited to one or two items per instrument.

Interpretation of Table 2 should be considered alongside Table 3, which documents patterns of construct drift across caregiver assessment tools. Several instruments—notably CNST-11 and MYLOH—primarily assessed caregiver strain, dyadic stressors, or caregiving preparedness rather than explicitly eliciting caregiver-defined/centered support needs. While such tools play an important role in screening for risk or vulnerability, they do not directly populate caregiver support need domains, underscoring the conceptual distinction between screening for stressors and assessing actionable caregiver-defined needs.

### 3.5. Sensitivity Analysis: Excluding CHAT and CHAT-D

Table 3 highlights that construct drift most commonly occurred through stressor screening and preparedness/capability framing, underscoring why “needs” labels cannot be assumed to reflect caregiver-defined support needs. As a sensitivity check, Table 2 counts were recalculated excluding CHAT and CHAT-D, given their conversational format and short pragmatic format. With these tools removed, the denominator decreased from 20 to 18 instrument entries. Domain proportions increased modestly (by 3–6 percentage points) across most domains; however, the relative ranking of domains remained unchanged, and patterns of under-representation (notably autonomy and spiritual/existential support) persisted. This confirms the robustness of the domain coverage findings.

## 4. Discussion

This rapid scoping review synthesized evidence from 43 studies describing 17 unique caregiver needs’ assessment instrument families and examined how caregiver needs are conceptualized, operationalized, and positioned for use in practice. By distinguishing caregiver-defined support needs from related but distinct constructs such as burden, strain, preparedness, and care-recipient monitoring, this review extends prior work by mapping the domains assessed in caregiver needs instruments and analyzing how caregiver “need” is defined and operationalized within existing tools. The findings highlight areas of alignment, reveal persistent gaps, and surface features that may shape the potential for these instruments to inform caregiver-centered care pathways in real-world health and social care systems.

### 4.1. Conceptual Weaknesses in How Caregiver “Need” Is Defined

Across the reviewed literature, caregiver “need” was seldom explicitly conceptualized [42]. Consistent with prior reviews, most instruments relied on implicit or atheoretical conceptualizations of need, typically operationalized as met/unmet items, problem lists, or proxy indicators such as burden, stress, or self-efficacy [27,42]. This aligns with Bangerter et al. [28] who noted the absence of a shared conceptual foundation in caregiver needs’ assessment. Bradshaw’s [93] typology of normative, felt, expressed, and comparative need appeared only occasionally, and largely as a classificatory reference rather than as a guiding analytical framework.

While Bradshaw’s [93] taxonomy remains useful for explaining why estimates of need vary depending on whether needs are defined by professionals, caregivers, or service use, it sits uneasily within contemporary caregiver-centered and rights-based approaches.

Normative need privileges professional assessment and expressed need risks equating silence with absence of need, despite evidence that caregivers may withhold needs due to duty, guilt, stigma, or lack of trust in services. McCabe’s *Hearing Their Voice* [94] illustrates this tension, highlighting that unexpressed needs often reflect social, cultural, and relational constraints rather than genuine adequacy.

Taken together, these patterns indicate that caregiver problems are often mistaken for caregiver needs. This conceptual misalignment weakens the validity of many instruments and limits their usefulness for planning responsive, caregiver-centered support.

### 4.2. Construct Drift and Its Implications for Caregiver-Centered Practice

A central contribution of this review is the identification of consistent patterns of construct drift across caregiver needs’ assessment instruments (Table 3). Several widely used tools assessed constructs adjacent to need, such as caregiver strain, dyadic stressors, or caregiving capability, without explicitly eliciting caregiver-defined support needs. For example, instruments such as CNST-11 [56] primarily function as stressor screening tools, while MYLOH [84,85] focuses on preparedness and confidence in managing care-recipient health. Although such tools are valuable for identifying risk or vulnerability, they do not directly capture what caregivers themselves identify as needing support, and are therefore less able to guide actionable care planning.

This pattern has important implications for practice. When instruments infer needs from burden or strain, caregivers’ priorities may be misinterpreted, potentially reducing opportunities for shared decision-making and tailored support. Moreover, reliance on proxy constructs risks perpetuating a deficit-oriented framing of caregiving, rather than recognizing caregivers as partners with legitimate, self-defined needs that warrant proactive and responsive system support.

### 4.3. Domain Patterns Reveal Enduring Gaps in Caregiver Needs’ Assessment

Mapping caregiver-defined support needs across instruments (Table 2) revealed substantial variation in domain coverage. Information, communication, and navigation support was the most consistently assessed domain, reflecting recognition that caregivers require guidance not only about illness management but also about navigating complex health and social care systems. Emotional and psychological support needs were also frequently included, though often restricted to measures of stress or distress rather than identity change, role transitions, anticipatory grief, or long-term adaptation.

Notably, autonomy, identity, and life participation needs, as well as spiritual, cultural, and existential needs, were among the least consistently assessed domains. This mirrors critiques of biomedical and task-oriented approaches, which privilege functional and task-oriented aspects of care while marginalizing relational, cultural, and meaning-making dimensions of caregiving [95]. The limited representation of these domains reduces the applicability of existing instruments in culturally diverse, Indigenous, palliative, and end-of-life contexts, where caregiving is deeply embedded in moral responsibility, identity, and relational continuity [24,26,96].

### 4.4. Why Caregiver Needs Tools Struggle to Translate into Care Pathways and EMRs

Beyond conceptual issues, this review highlights a persistent gap between caregiver needs’ assessment as a research activity and its use as a routine component of care delivery. Implementation-relevant properties, including administration time, training requirements, workflow fit, scoring interpretation, and linkage to follow-up actions, were inconsistently reported [97,98]. Even tools frequently cited as best practice, such as CSNAT, experience barriers to system-level uptake, including documentation burden, workflow disruption, and limited integration into EMRs [99,100].

Few instruments were explicitly designed to support longitudinal reassessment, interdisciplinary communication, or seamless documentation within EMRs. As a result, caregiver needs’ assessment remains episodic, siloed, and weakly connected to care planning and service coordination. These limitations signal an opportunity to reposition caregiver needs’ assessment as an integrated care pathway process, rather than a stand-alone measurement task.

### 4.5. Limitations

This rapid scoping review involved methodological streamlining, including restriction to peer-reviewed English-language literature, exclusion of grey literature, and single reviewer title/abstract screening following calibration. These choices may have reduced identification of unpublished tools, implementation manuals, or locally developed needs’ assessments used in practice. However, our primary conclusions are unlikely to be substantially affected because the review focus was on instrument content and conceptualization of “need” using item-level analysis, and the dominant pattern observed—frequent reliance on proxy constructs and limited workflow/EMR readiness—was consistent across multiple countries, settings, and instrument families represented in the included peer-reviewed literature. In addition, backward/forward citation searching and an update search supported identification of widely cited instruments and newer tool development reports.

## 5. Implications for Practice and Policy

The findings from this review have important implications for the design, selection, and implementation of caregiver needs’ assessment tools within health and social care systems. This review highlights the need for a clearer caregiver-centered approach to tool development and implementation. In this context, a caregiver-centered lens refers to assessment that prioritizes caregivers’ own articulated support needs, acknowledges unmet or unspoken needs, and positions caregivers as partners in care planning rather than as secondary to patient outcomes.

Accordingly, caregiver needs’ assessment must shift beyond reliance on proxy indicators such as burden, stress, or preparedness, and instead directly elicit the support caregivers require to sustain their role. Instruments that conflate impacts with needs risk misaligning services with priorities that matter to caregivers. Health and social care systems should therefore prioritize tools that explicitly ask caregivers what support they need, attend to needs that may not be verbally expressed, and facilitate shared decision-making and actionable care planning.

Second, assessment tools must be designed with workflow integration in mind. Instruments intended for routine use should be concise and designed to fit workflows, whether self-administered, clinician-facilitated, or completed collaboratively, with clear guidance on interpretation, follow-up, and referral. Embedding caregiver assessment prompts and documentation fields within EMRs can support continuity across care settings, enable interdisciplinary communication, and normalize caregiver inclusion as part of standard care processes. Without such integration, even well-validated tools are unlikely to be used consistently.

Third, caregiver needs’ assessment should function as an ongoing, longitudinal process rather than a single point-in-time measure. Caregiver roles, capacities, and support needs shift as health conditions progress, care transitions occur, and responsibilities intensify or diminish. Health and social care systems therefore require mechanisms for repeated assessment, timely responsiveness to change, and tracking of persistent or emerging unmet needs. This approach necessitates tools with sensitivity to change and actionable thresholds that can prompt reassessment, referral, or intervention within a care pathway.

Fourth, future development and implementation of caregiver assessment tools must explicitly integrate equity considerations. The findings from this review show that domains related to identity, autonomy, spirituality, and meaning were seldom assessed, reflecting a broader pattern in which caregiver needs are approached through a biomedical and task-focused lens. When assessment frameworks are developed without attention to structural conditions, such as income, language, immigration status, rurality, and access to services, tools risk overlooking caregivers whose needs are shaped by social position and who face greater barriers to support.

Embedding equity means recognizing that caregiver experiences and support needs are not uniform; they vary across socioeconomic contexts, care trajectories, and the distribution of labour within families. Tools that do not account for these differences may inadvertently privilege caregivers who are resourced, literate in health systems, or comfortable requesting help. Equity-oriented assessment should therefore help surface needs that remain invisible, unspoken, or constrained by structural barriers, and support proactive outreach and navigation for caregivers at greatest risk of unmet need.

Finally, policy efforts should support caregiver needs’ assessment as a core component of integrated care. This includes providing guidance on tool selection, investing in training for providers, establishing clear referral pathways, and aligning funding and accountability mechanisms to ensure that identified needs lead to meaningful support. Without system-level commitment, caregiver needs’ assessment risks remaining an underutilized measurement exercise rather than a lever for improving caregiver wellbeing and care system performance.

## 6. Conclusions

This rapid scoping review highlights a persistent disconnect between the growing recognition of family caregivers as essential partners in care and the limited capacity of existing caregiver needs’ assessment tools to support their systematic inclusion in care delivery. Although many instruments are available, most remain oriented toward research or episodic screening and rely heavily on proxy indicators such as burden or strain, rather than eliciting caregiver-defined support needs that can inform care planning, navigation, and follow-up. As a result, caregiver needs’ assessment is rarely embedded within routine workflows, interdisciplinary care pathways, or EMRs, constraining its utility for health system integration.

These findings have direct relevance to the Alberta Caregiver Strategy, which calls for consistent caregiver identification, proactive needs’ assessment, seamless navigation, and coordinated supports across care settings. Without workflow-ready, digitally enabled assessment tools, these strategic commitments are difficult to operationalize in practice. Similarly, the results reinforce priorities articulated in the Caregiver-Centered Care Competency Framework, particularly the need for health and social care providers to recognize caregivers, engage them as partners, assess their needs longitudinally, and translate assessment findings into concrete actions and supports. Tools that do not align with these competencies risk perpetuating caregiver invisibility and limiting providers’ ability to enact caregiver-centered practice.

Advancing caregiver-centered care in Alberta and beyond will require a deliberate shift from viewing caregiver needs’ assessment as a measurement exercise to positioning it as a core system function. Assessment tools must be co-designed with caregivers, responsive to diverse cultural and social contexts, and explicitly designed to integrate with care pathways, documentation systems, and navigation infrastructure. When embedded within routine practice and supported by trained providers, caregiver needs’ assessment can serve as a critical mechanism for operationalizing caregiver-centered competencies, strengthening continuity of care, and improving system performance.

In the absence of such integration, caregiver needs will continue to be acknowledged rhetorically but addressed inconsistently in practice. Aligning caregiver needs’ assessment with provincial strategy and competency-based care offers a tangible pathway to move from recognition to action, thereby ensuring that caregivers are not only seen but systematically supported as partners in care.

## Figures and Tables

**Table 1:** Caregiver assessment instruments: scope, content, and administration.

Instrument	Items	Domain Description (Person-Centered Caregiver Lens)	Administration Method
* Caregiver Checklist for Primary Care [51]	7 items	Brief checklist to surface caregiver-reported concerns and support needs relevant to primary care (e.g., understanding conditions/medications, personal care tasks, coping/stress, financial/legal issues, and arranging services/care coordination). Intended to prompt focused discussion, referral, and follow-up rather than comprehensive assessment.	Self-administered or completed collaboratively with primary care staff (paper/electronic), ideally pre-visit or at the visit to guide follow-up discussion
Caregiver Needs and Resources Assessment (CNRA) [52]	36	Assesses caregiver-identified needs and resources related to physical wellbeing, psychological needs, role conflict, social support, and caregiving demands, alongside personal strengths and resources (e.g., spirituality, self-efficacy, family and community supports) that support caregivers’ capacity to sustain care and maintain wellbeing.	Self-administered; trained interviewers supported completion for caregivers with literacy needs
Caregiver Needs Assessment (CNA) [53]	18	Identifies caregiver-reported needs for information, practical support, emotional and spiritual support, relational support, communication with health professionals, involvement in decision-making, and access to services, reflecting caregivers’ priorities for partnership and support.	Self-administered; social workers involved for literacy support
Caregiver Needs Scale (CNS) [54]	20	Captures caregiver-identified support needs related to accessing information, services, social support, childcare, and financial resources, with a focus on enabling caregivers to meet family and caregiving responsibilities.	Self-administered
Caregivers’ Aspirations, Realities, and Expectations Tool (CARE Tool) [55]	15 scored assessment areas comprising 104 main questions, many with structured sub-questions and probes	Comprehensive assessment of caregivers’ lived experience, including caregiving tasks, service use, health and wellbeing, financial impact, family relationships, role strain, and future planning, supporting holistic understanding of caregivers’ circumstances and goals.	Administered by trained home care practitioners (e.g., nurses, social workers, rehabilitation professionals)
Carer Need Screening Tool (CNST-11) [56]	11	Screens for caregiver-reported challenges and caregiving-related stressors affecting wellbeing and the caregiving relationship, to flag areas for follow-up discussion and support; primarily a stressor/strain screening tool rather than an instrument that elicits caregiver-defined support needs.	Self-administered
Carer Support Needs Assessment Tool (CSNAT) [57,58,59,60,61,62,63,64,65,66,67,68]	14–15	Explicitly elicits caregiver-defined support needs, distinguishing needs that enable caregiving from needs that support caregivers’ own health, wellbeing, emotional coping, and social and financial circumstances, forming the basis for shared action planning.	Self-administered; face-to-face or telephone interviews
Carers’ Alert Thermometer (CAT) [69,70]	11 (+optional EOL item)	Rapid identification of caregiver-perceived unmet needs across practical care, emotional and spiritual support, information, respite, emergency preparedness, decision-making involvement, and balancing caregiving with personal needs.	Completed collaboratively with staff or advisors
** Care Partner Hospital Assessment Tool (CHAT; CHAT-D) [38,71,72,73]	Brief structured prompts (~10–15 items, conversational)	Brief, structured conversational prompts to elicit caregiver-defined support needs and priorities during hospitalization and discharge planning (e.g., information/training needs, coping support, role expectations, and post-discharge coordination). Designed to guide timely follow-up and shared planning rather than to provide a comprehensive multidomain assessment.	Clinician-facilitated conversation in hospital (and/or transitional care settings); documentation intended to inform discharge planning and follow-up
Family Inventory of Needs (FIN) [74,75]	20	Assesses caregivers’ perceptions of the importance of specific needs and the extent to which those needs are met by health services, highlighting gaps in person-centered support and communication.	Self-administered
Family Needs Assessment (FNA) [76,77]	59–76	Multidimensional assessment of family and caregiver needs related to caregiving, autonomy, social participation, emotional wellbeing, financial security, access to services, and future planning, reflecting family-centered and caregiver-inclusive care.	Self-administered; online or paper-based
Family Needs Questionnaire (FNQ) [78,79]	37–40	Identifies caregiver-reported needs for information, emotional and instrumental support, professional and community support, and involvement in care, emphasizing partnership with providers and social connectedness.	Self-administered
Family Needs Survey (FNS) [80,81,82]	34	Assesses caregivers’ informational, social, and financial needs, including needs related to explaining the caregiving situation to others, supporting social inclusion and shared understanding.	Self-administered
interRAI Family Carer Needs Assessment [83,84]	87	Systematic assessment of caregiver wellbeing, needs, supports, and caregiving experience within a standardized care framework, intended to inform coordinated, caregiver-inclusive care planning.	Self-administered
Managing Your Loved One’s Health (MYLOH) [85,86]	29	Identifies caregiver needs related to understanding and managing the care recipient’s health, recognizing changes, making healthcare decisions, and accessing support, emphasizing preparedness and confidence to sustain care.	Self-administered
*** PCQN caregiver needs assessment [87]	17	Brief assessment of caregiver-reported needs related to distress, knowledge and skills, emotional support, health and wellness, and practical assistance, designed to support timely, person-centered clinical response.	Brief self-report during visits or by phone; often reviewed with a team member
*** PHN caregiver assessment tool [88]	75	Assesses caregiver needs across health, emotional coping, financial and housing stability, family relationships, social supports, and skills training, supporting comprehensive, relationship-based public health nursing practice.	Clinician-led interviews
Supportive Care Needs Survey–Partners and Caregivers (SCNS-P&C) [89,90,91]	44–45	Measures caregiver-reported supportive care needs related to healthcare services, emotional wellbeing, work and social roles, and information, highlighting areas where systems can better support caregiver quality of life.	Self-administered
Unmet Needs Assessment Tool [92]	57	Identifies caregiver-reported unmet needs related to health and care, employment, information, emotional and social support, and caregiver burden, supporting needs-based follow-up and referral.	Self-administered; digital

* Caregiver Checklist for Primary Care: Brief, pragmatic checklist developed for primary care to surface caregiver concerns and priorities and prompt focused discussion and follow-up, rather than comprehensive assessment. ** CHAT: A brief, clinician-facilitated screening and clinical decision-support tool designed to identify caregiver priorities, training needs, and support requirements during hospitalization and care transitions, rather than to provide a comprehensive needs’ assessment. *** Tools are newly developed instruments without formal names given. “PCQN” was a clinical caregiver needs’ assessment tool developed as the first phase to improve the quality of care for patients with serious illness and their families supported by the Palliative Care Quality Network (PCQN); “PHN” was a proposed assessment tool for use by public health nurses (PHN).

**Table 2:** Caregiver-defined support needs domains and instrument coverage (needs for support items only).

Category	Example Support Need Items	Instruments with ≥1 Needs for Support Item in This Domain **	n (%) *
Caregiver health & self-care support needs	“Do you need more support looking after your own health?” (CSNAT)	CSNAT; PCQN; PHN; SCNS-P&C; CAT; CNA; CARE Tool; interRAI	8 (40%)
Emotional & psychological support needs	“Do you need more support dealing with your feelings and worries?” (CSNAT)	CSNAT; FNQ; SCNS-P&C; CAT; CNA; CARE Tool; PHN; PCQN; interRAI	9 (45%)
Information, communication & navigation support needs	“Understanding relative’s illness” (CSNAT); “Who to call in an emergency…” (CAT)	CSNAT; CNA; CAT; FNQ; SCNS-P&C; FNS; FNA; PCQN; PHN; interRAI; CARE Tool	11 (55%)
Practical & instrumental support needs	“Do you need more support with financial, legal, or work issues?” (CSNAT)	CSNAT; FNQ; FNA; CNA; CAT; CNS; PHN; PCQN; SCNS-P&C; CARE Tool	10 (50%)
Social & relational support needs	“Support from relatives/manage change in relations” (CNA)	CNA; SCNS-P&C; FNQ; FNS; FNA; CSNAT; PHN; CARE Tool	8 (40%)
Autonomy, identity & life participation support needs	“Finding more time for myself” (FNS); “participating in social occasions…” (FNA)	FNS; FNA; CSNAT; FNQ; CNA; interRAI; CARE Tool	7 (35%)
Spiritual, cultural & existential support needs	“Your beliefs or spiritual concerns” (CSNAT); “emotional or spiritual care…” (CAT)	CSNAT; CNA; CAT; FNA; FNS; PCQN; SCNS-P&C	7 (35%)

* Denominator is 20 instrument entries (including distinct adaptations or context-specific versions). ** Domain coverage reflects items explicitly framed as caregiver needs for support. Items reflecting caregiving capability or preparedness (e.g., MYLOH), care-recipient monitoring, stressor/strain screening (e.g., CNST-11), or wellbeing impacts without an explicit request for support were coded separately as construct drift and are not included in domain coverage counts.

**Table 3:** Construct drift categories identified in caregiver assessment instruments.

Construct Category *	Description	Example Item(s) from Extracted Tools	Examples of Tools Where Observed
Caregiving capability/preparedness	Knowledge/skill confidence, decision readiness, ability to manage care tasks	“I understand what his/her problems are with medical issues.” (MYLOH)	MYLOH; PCQN (knowledge/skills items); CNRA (resources/self-efficacy items; resources ≠ needs)
Care-recipient monitoring/management	Vigilance, recognizing deterioration, symptom tracking, behaviour observation	“I can tell when there are new or rapidly worsening changes in mood/behaviours.” (MYLOH)	MYLOH; CARE Tool (task-heavy sections); CNRA (care-recipient status items)
Stressor/strain screening	Flags burden, dyadic stressors, or relationship dysfunction rather than eliciting support needs	“Multifaceted challenges/caregiving stressors…” (CNST-11 factor description)	CNST-11; Unmet Needs Assessment Tool (burden domain)
Care-recipient symptom statements embedded in caregiver tools	Statements about the care-recipient that may drive caregiver needs but are not needs items themselves	“He/she is emotionally disturbed.” (CNRA)	CNRA; other mixed caregiver–recipient tools
Wellbeing impact statements (without support request)	Negative effects on health or life not framed as a request for help	“Problems with sex life.” (SCNS-P&C)	SCNS-P&C; CNRA (impact phrasing); others depending on wording

* These construct categories were retained to characterize how instruments operationalize “needs” in practice but were not counted toward needs-domain coverage unless the item explicitly elicited a caregiver support need.

## Data Availability

The datasets used and/or analyzed during the current study are available from the corresponding author on reasonable request.

## Supplementary Materials

The following supporting information can be downloaded at: https://www.mdpi.com/article/10.3390/ijerph23030300/s1, File S1: Search Strategy; File S2: Categorized domains of existing caregiver need items, explanation, and File S3: Item-level mapping of included caregiver needs assessment instruments to conceptual domains. Reference [101] is cited in the Supplementary Materials.
