# Supplementary material for: Mapping Caregiver Needs’ Assessment Tools for Family and Friend Caregivers: A Rapid Scoping Review
Source: Int J Environ Res Public Health. 2026 Feb 28;23(3):300. doi: 10.3390/ijerph23030300 (PMC13027153; doi:10.3390/ijerph23030300)
Supplement: Supplementary file 1 [file ijerph-23-00300-s001.zip › ijerph-4143714-supplementary S1-S3.pdf]

Supplementary Materials
S1: Search Strategy

CINAHL

Row	Searches	Results
1	XB (scale* or questionnaire* or assess* or instrument* or tool* or checklist* or measure*)	2,059,056
2	TI need*	109,135
3	XB (caregiver* or carer or "Informal caregiver*" or "unpaid caregiver" or "care partner*" or care-partner* or "family caregiver*")	88,567
4	S1 AND S2 AND S3	1,521

MEDLINE

Row	Searches	Results
1	(scale* or questionnaire* or assess* or instrument* or tool* or checklist* or measure*).tw	9,297,907
2	"need*".ti	194,464
3	(caregiver* or carer or "Informal caregiver*" or "unpaid caregiver" or "care partner*" or care-partner* or "family caregiver*").tw	117,042
4	1 and 2 and 3	2,020

Cochrane Library

Row	Searches	Results
1	(scale* or questionnaire* or assess* or instrument* or tool* or checklist* or measure*):ti,ab	1,122,846
2	"need*":ti	2,472
3	(caregiver* or carer or "Informal caregiver*" or "unpaid caregiver" or "care partner*" or care-partner* or "family caregiver*"):ti,ab	22,759
4	#1 AND #2 AND #3	22

PsycInfo

Row	Searches	Results
1	(scale* or questionnaire* or assess* or instrument* or tool* or checklist* or measure*).tw	2,109,803

2	"need*".ti	46,275
3	(caregiver* or carer or "Informal caregiver*" or "unpaid caregiver" or "care partner*" or care-partner* or "family caregiver*").tw	76,472
4	1 and 2 and 3	1,067

Health-Assessment and Psychological Instruments (HAPI)

Row	Searches	Results
1	(scale* or questionnaire* or assess* or instrument* or tool* or checklist* or measure*).tw	144,587
2	"need*".ti	910
3	(caregiver* or carer or "Informal caregiver*" or "unpaid caregiver" or "care partner*" or care-partner* or "family caregiver*").tw	3,956
4	1 and 2 and 3	31

Web of Science

Row	Searches	Results
1	(TI=((scale* or questionnaire* or assess* or instrument* or tool* or checklist* or measure*))) OR AB=((scale* or questionnaire* or assess* or instrument* or tool* or checklist* or measure*))	35,002,207
2	TI=("need*")	864,920
3	((TI=("need*")) AND TI=((caregiver* or carer or "Informal caregiver*" or "unpaid caregiver" or "care partner*" or care-partner* or "family caregiver*"))) OR AB=((caregiver* or carer or "Informal caregiver*" or "unpaid caregiver" or "care partner*" or care-partner* or "family caregiver*"))	219,517
4	#1 AND #3 AND #4	3,411

S2: Categorized domains of existing caregiver need items, explanation, and illustrative examples

Domains	Explanation	Illustrative Examples
Health and physical needs	Caregivers’ own physical health, well-being, and ability to manage care recipients’ physical needs safely and effectively	Managing caregiver’s sleep, stress, and chronic conditions; supporting care recipient’s ADL/IADL, medication administration, nutrition, mobility, and safety; access to healthcare professionals for both the care recipient and caregivers; timely diagnosis; sexuality/intimacy
Psychological and emotional needs	Support for coping with caregiving stress, emotional burden, anticipatory grief, and maintaining mental health	Emotional support from peers or professionals, counseling, support groups, coping strategies for stress, managing feelings of isolation, dealing with anxiety or depression, anticipatory grief (feelings of loss) for both caregiver and care recipient, support after caregiving ends
Informational and knowledge needs	Access to relevant knowledge about the care recipient’s condition, caregiving tasks, available resources, and self-care strategies	Education on disease symptoms and progression, prognosis, treatment options; guidance on legal issues and advance directives; caregiver training; communication skills; one-stop access to community and health services;
Practical and instrumental support needs	Tangible help that enables caregivers to manage day-to-day responsibilities for themselves and the care recipient	In-home assistance (housework, meals, transport), respite care (day or overnight), financial and legal support (especially with care-at-home costs), care coordination, flexible services, access to assistive devices and communication technology, navigating healthcare and social systems, support with problem-solving and decision-making, childcare
Social and relational needs	Maintaining supportive interpersonal relationships, involvement of family/friends, and social integration	Family and friend support and involvement in care, shared responsibility, peer support, social networks, maintaining relationships and communication, societal understanding, opportunities to share experiences with other caregivers, fostering mutuality with care recipient (emotional connection)
Autonomy and lifestyle needs	Preserving the caregiver’s independence, personal time, and participation in leisure, work, and other meaningful activities	Personal time, leisure activities, work-life balance, maintaining hobbies, space for self-care, opportunities for respite, role adjustment (e.g., parent-child role reversal)
Spiritual, Cultural, and Existential Needs	Support for meaning-making, spiritual coping, cultural considerations, and recognition of the caregiver as a person with their own values and beliefs	Spiritual or faith-based support, culturally tailored care and services (to address cultural differences and language barriers), family-centred care, empowerment, dignity, being recognized as a person with unique needs, addressing beliefs and values in care planning, exploring knowledge of support

		system available in ancestral country of origin, support for young caregivers, female and male react differently to the care role, children and spouse react differently to the care role

S3. Item-level mapping of included caregiver needs assessment instruments to conceptual domains

Tool name	Domain							
	Health and Physical Needs	Psychological and Emotional Needs	Informational and Knowledge Needs	Practical and Instrumental Support Needs	Social and Relational Needs	Autonomy and Lifestyle	Spiritual, Cultural, and Existential Needs	No Domain
Caregiver Needs and Resources Assessment (CNRA)	1 - Taking care of him/her deprives me of sleep.<br>2 - Taking care of him/her brings me poor health.<br>3 - Taking care of him/her brings me poor appetite.<br>34 - I can set a health plan and goal for myself.	8 - His/her problematic behavior annoys me.<br>9 - He/she is emotionally disturbed.<br>10 - Taking care of him/her makes me confused.<br>11 - Taking care of him/her makes me lose interest in everything.<br>12 - I often have negative emotions (e.g., anxiety, sadness, frustration, annoyance).<br>25 - I can face and resolve the difficulties encountered during caregiving.	22 - I am clear about the responsibilities as a caregiver.<br>23 - I know how to take care of him/her appropriately.<br>24 - I can effectively resolve the problems in caregiving.	4 - Taking care of him/her has impact on my job.<br>26 - I think the resources/services provided by my community for caregivers are satisfactory.<br>27 - I think the resources/services provided by my community for care recipients are satisfactory.	5 - Taking care of him/her brings me conflict with the family member.<br>6 - Taking care of him/her affect my family life.<br>13 - The responsibility of caregiving estranges me from my friends.<br>14 - I have less opportunities in keeping in touch with my friends.<br>28 - My family can help me in	15 - Taking care of him/her restricts my participation in social activities.<br>20 - I accept my identity as a family caregiver.<br>21 - No matter how hard it could be, I have the responsibility to take care of him/her.<br>35 - I work out regularly.<br>36 - I learn new skills or establish new habits.	My spiritual/ religious belief supports me in taking care of him/her.<br><br>Taking care of him/her is meaningful.<br><br>My faith/ religious belief helps me in understanding the difficulties in caregiving.<br><br>Taking care of him/her helps me in understand	7 - He/she has poor cognitive ability.

		31 - He/she always makes me angry. (r)			tackling the difficulties in caregiving.<br>29 - I have good relationship with my family.<br>30 - When I encounter difficulties, I will at least have one family member from whom I can seek advice or support.<br>32 - The trust between me and him/her declines. (r)<br>33 - I argue with him/her on trivial things.		ing the meaning of life.	
Family Needs Questionnaire (FNQ)	11 - To have complete information on the patient's physical problems<br>22 - To get enough rest or	31 - To discuss my feelings about the patient with someone who has gone through the same	1 - To be shown that medical, educational or rehabilitation staff respect the patient's needs and wishes<br>4 - To be told	20 - To have help keeping the house<br>21 - To have help from other members of the family in taking care of the	27 - To have my partners or friends understand how difficult it is for me<br>28 - To have other family	23 - To get a break from my problems and responsibilities<br>24 - To spend time with my friends<br>25 - To pay		2 - To be told daily what is being done with or for the patient *(too operational/monitoring focused)*<br>3 - To give my

	sleep	experience<br>33 - To be reassured that it is usual to have strong negative feelings about the patient<br>34 - Help getting over my doubts and fears about the future<br>35 - Help remaining hopeful about the patient's future<br>36 - Help preparing for the worst	about all changes in the patient's medical status<br>5 - To be assured that the best possible medical care is being given to the patient<br>6 - To have explanations from professionals given in terms I can understand<br>7 - To have my questions answered honestly<br>10 - To have complete information on the medical care of traumatic injuries<br>12 - To have complete information on the patient's problems in thinking<br>13 - To have complete information on drug and alcohol	patient<br>18 - To have enough resources for the patient<br>19 - To have enough resources for myself or the family	members understand the patient's problems<br>29 - To have the patient's friends understand his/her problems<br>30 - To have the patient's employer, coworkers or teacher understand his/her problems<br>32 - To discuss my feelings about the patient with other friends or family	attention to my own needs, job or interests		opinions daily to others involved in the patient's care, rehabilitation or education<br>8 - To be shown that my opinions are used planning the patient's treatment, rehabilitation or education<br>9 - To have a professional to turn to for advice or services when the patient needs help<br>15 - To be shown what to do when the patient is upset or acting strange<br>17 - To have help in deciding how much to let the patient do by himself/herself<br>37 - To be encouraged to ask others to help out

			problems and treatment<br>14 - To be told how long each of the patient's problems is expected to last<br>16 - To have information on the patient's rehabilitative or educational process					
Caregiver Needs Scale (CNS)			need for information	need for community services<br>need for financial support	need for social support			
Carer Support Needs Assessment Tool (CSNAT)	8 - Looking after your own health<br>9 - Equipment to help care for your relative	6 - Dealing with your feelings and worries	1 - Understanding relative's illness<br>7 - Knowing who to contact if concerned<br>13 - Knowing what to expect in the future	4 - Financial, legal, or work issues<br>5 - Providing personal care<br>12 - Practical help in the home	11 - Talking with relative about their illness	2 - Time for yourself in the day<br>14 - Getting break from caring overnight	10 - Your beliefs or spiritual concerns	Health and Physical Needs – 2 items (8,9)<br><br>Psychological and Emotional Needs – 1 item (6)<br><br>Informational and Knowledge Needs – 4 items (1,7,11,13)<br><br>Practical and Instrumental Support Needs – 3 items (4,5,12)<br><br>Social and

								Relational Needs – 0 items (no purely relational question) or 1??? idk about 11<br><br>Autonomy and Lifestyle Needs – 2 items (2,14)<br><br>Spiritual, Cultural, and Existential Needs – 1 item (10)
Supportive Care Needs Survey – Partners and Caregivers (SCNS-P&C)	14 - Reduce stress for patient<br>15 - Look after own health<br>16 - Pain control for patient<br>18 - Fertility problems in patient<br>35 - Changes in patient's body<br>36 - Problems with sex life	17 - Fears about patient deterioration<br>31 - Concerns about recurrence<br>37 - Emotional support for self<br>38 - Emotional support for loved ones<br>39 - Feelings about death<br>40 - Not acknowledging impact of caring<br>41 - Recovery not as expected	1 - Information carer needs<br>2 - Information prognosis<br>3 - Information support services<br>4 - Information alternative therapies<br>5 - Information patient physical needs<br>6 - Information for decision making<br>7 - Best medical care patient<br>8 - Access local health services<br>9 - Involved in	19 - Practical caring tasks<br>20 - Accessible hospital parking<br>22 - Life/work changes for carer<br>23 - Financial/government support<br>24 - Insurance for patient<br>25 - Access legal services<br>12 - Case manager coordinated services	26 - Communicate with patient<br>27 - Communicate with family<br>28 - Support from family<br>29 - Talk to other cancer carers<br>30 - Discuss cancer at work/socially<br>32 - Impact on relationship with patient	21 - Changes to patient's life/work<br>34 - Balancing own and patient's needs	43 - Own spiritual beliefs<br>44 - Meaning in patient's illness	

			patient care<br>10 - Discuss concern with doctor<br>11 - Doctor coordinated care<br>13 - Complaints regarding care addressed<br>33 - Understand patient experience<br>42 - Decision making in uncertainty<br>45 - The opinion to influence decisions in the treatment of the patient's illness					
Managing Your Loved One's Health (MYLOH)	1.A - I understand what his/her problems are with memory (ex. remembering, planning, making decisions)<br>2.A - Right now, I can deal with day-to-day problems he/she has with memory (ex.	1.B - I understand what his/her problems are with mood/behaviors (ex. anger, sadness, irritation, poor sleep)<br>2.B - Right now, I can deal with day-to-day problems he/she has with mood/behavior	6.A - I can tell when there are new or rapidly worsening changes in his/her memory (ex. remembering, planning, making decisions)<br>7.A - When new or rapidly worsening changes happen, I know	9.D - I know what to do if I have a personal crisis and cannot provide care or help with care as I usually do<br>8.C - In helping with health care decisions, I understand the responsibilities of a person who has a Power of Attorney for Medical		2.D - Right now, I can deal with day-to-day problems he/she has with self-care (ex. eating, dressing, showering, using the toilet)<br>9.B - I am taking care of myself so that I can continue to care for him/her (ex. I take a break when needed)		

	remembering, planning, making decisions)<br>1.C - I understand what his/her problems are with medical issues (ex. illnesses, pain, headaches)<br>2.C - Right now, I can deal with day-to-day problems he/she has with medical issues (ex. illnesses, pain, headaches)<br>3.A - I know, or can get information about what medications his/her health care provider recommends (prescription and non-prescription)<br>3.B - I know, or can get information about what dose, when and how these	s (ex. anger, sadness, irritation, poor sleep)<br>6.B - I can tell when there are new or rapidly worsening changes in his/her mood/behaviors (ex. anger, sadness, irritation, poor sleep)<br>7.B - When new or rapidly worsening changes happen, I know what I can deal with on my own<br>8.A - In helping with health care decisions, I understand what would be important to the person I care for	what to watch for and what to report to his/her healthcare provider<br>7.C - When new or rapidly worsening changes happen, I know when to contact his/her health care provider<br>7.D - When new or rapidly worsening changes happen, I know which health care provider I should contact (ex. doctor, nurse, pharmacist)<br>9.C - When I need help with caregiving, I know how to get it	Decision Making<br>8.B - In helping with health care decisions, I understand how to speak up on his/her behalf				

	medications should be taken (ex.10 mg tablet twice a day)<br>3.C - I know, or can get information about what conditions these medications are used for (ex. blood pressure, blood sugar, dementia)<br>4 - I watch to be sure that he/she takes medications correctly and provide help when needed<br>5 - If I have concerns about his/her medications (ex. I worry about the safety or value of what is prescribed) I tell the clinician about them<br>6.C - I can tell when there are new or rapidly worsening changes in his/her medical issues (ex.							

	illnesses, pain, headaches)<br>6.D - I can tell when there are new or rapidly worsening changes in his/her self-care (ex. eating, dressing, showering, using the toilet)<br>7.E - When new or rapidly worsening changes happen, I know when I need immediate assistance and should call 911 or other emergency medical help							
Unmet Needs Assessment Tool	(1) health and care	(4) emotional support	(3) information and knowledge<br>(6) caregiver burden		(5) social support	(2) employment		
interRAI Family Carer Needs Assessment								
Caregiver Needs Assessment (CNA)	3 - Problematic behaviors	11 - Reassurances<br>16 - Psychological support	1 - Information on the disease<br>5 - Knowledge of future problems<br>12 -	2 - Caretaking tasks<br>4 - Help in caregiving<br>6 - Financial support	8 - Manage change in relations<br>9 - Support from relatives	14 - Privacy	17 - Spiritual support	18 - Help in homework

			Communication with health staff	15 - Community services	13 - Sharing the experience of others (peer or social support)<br>10 - Involvement in decisions			
Family Needs Assessment (FNA)	23. Having appropriate vision and eye care<br>44. Having appropriate dental care<br>53. Getting a full night's sleep<br>67. Preventing substance abuse and other addictions (e.g., alcohol, drugs)<br>61. Having healthy life style (such as healthy diet/exercising)<br><br>13. Going to bathroom (as in teaching or going themselves or..)<br>24. Giving medications	6. Participating in goal-setting to enhance family members' learning<br>31. Enhancing each family member's self-esteem<br>69. Teaching social and emotional skills<br>74. Teaching communication skills<br>20. Managing stress<br>54. Having a break from caretaking (such as respite care)	7. Having educational services where my child(ren) are making progress<br>8. Having a clear understanding of each family member's strengths and needs<br>5. Feeling supported by professionals at the time of learning about my child(ren)'s disability<br>37. Helping all family members to know how to respond to questions about disability<br>70. Getting	11. Paying basic needs (such as food, house, clothing)<br>22. Paying school fees and/or child care (babysitter)<br>43. Saving money for the future<br>52. Getting or keeping a job<br>33. Paying for special therapies or equipment for my child<br>60. Applying for government benefits and addressing government benefit denials<br>62. Having appropriate transportation<br>29. Having access to	45. Getting child care<br>16. Getting new childcare 51. Dealing with challenges related to all family members<br>63. Using technological communications (such as email, Facebook) to connect socially with others<br>59. Providing supports to include all members of my family in family	3. Participating in preferred indoor community recreational activities (e.g., movies, concerts, art classes)<br>14. Participating in preferred outdoor community recreational activities (e.g., swimming, playing ball, playing in the parks)<br>25. Going on family vacations<br>55. Participating in social occasions with friends, co-workers, or others<br>18. Having appropriate extracurricular/ho	32. Understanding my family members' challenges within my family's spiritual beliefs<br>21. Teaching my child(ren) about spiritual beliefs<br>10. Having a spiritual community that includes my child (ren)	56. Starting a new school year<br>26. Helping my family members make friends<br>30. Solving problems together<br>27. Moving within the same community or to a different community<br>77. Helping with homework<br><br>58. Making changes in services when necessary, even when professionals disagree<br>48. Teaching safety in the home and other places<br>38. Planning for

	34. Having appropriate care for hearing related needs<br>1. Monitoring health conditions (having a regular doctor/health checks)<br>28. Knowing when my children) is making progress		information necessary to make sound decisions about services<br>66. Knowing and acting on my child(ren)'s educational rights<br>73. Feeling informed and helped by teachers about the improvement and the difficulties of my child(ren)35. Getting regular and special resources (such as technology equipment and materials, adapted switches, special foods) needed by family members<br>2. Teachin he daily ao activite if ng ning, brushing teeth, dressing, eating (is this typo?)	necessary services, such as speech therapy, physio/physical therapy, orientation and mobility, occupational therapy, audiology, and nursing care<br>71. Feeling supported by professionals to manage the difficulties associated with daily living.<br>49. Monitoring services to make sure that they are beneficial<br>68. Ensuring that home and community settings are accessible	activities<br>15. Helping my family members (e.g., neighbors, friends) in socializing with others<br>4. Helping others (e.g., neighbors, friends) in knowing how to socialize with my family members<br>12. Coordinating medical care among two or more physicians<br>50. Being flexible as a family in making changes when they are needed<br>42. Having support from other families who have a child with	liday care<br>36. Doing relaxing things/activities at home		my child(ren)'s successful transition from preschool to primary school or from primary school to secondary school<br>17. Helping my child(ren) reach goals during every day routines<br>39. Teaching choice-making and problem-solving<br>76. Teaching my child(ren) about sexuality<br>72. Teaching appropriate behavior

			57. Teaching independent living skills (such as eating and dressing)<br>75. Teaching motor skills (e.g., riding a bike, walking, climbing stairs)		disabilities<br>64. Planning for the future after I'm no longer able to take care of my family members<br>46. Responding to negative situations and attitudes (e.g., bullying, teasing, staring) to all family members<br>47. Developing long-term goals for family members<br>41. Establishing close emotional bonds among members of the family<br>40. Having a trusting partnership			

					with professional s<br>19. Talking about feelings, opinions, and challenges with all members in my family			
Family Inventory of Needs (FIN)	3 - Feel that the health professionals care about the patient<br>9 - Feel there is hope<br>17 - Feel accepted by the health professionals	1 - Have my questions answered honestly<br>2 - Know specific facts concerning the patient's prognosis<br>4 - Be informed of changes in the patient's condition<br>5 - Know exactly what is being done for the patient<br>6 - Know what treatment the patient is receiving<br>7 - Have explanations given in terms that are understandabl	18 - Help with the patient's care<br>20 - Be told about people who could help with problems					

		e<br>8 - Be told about treatment plans while they are being made<br>11 - Know what symptoms the treatment or disease can cause<br>12 - Know when to expect symptoms to occur<br>13 - Know the probable outcome of the patient's illness<br>14 - Know why things are being done for the patient<br>15 - Know the names of health professionals involved in the patient's care<br>16 - Have information about what to do for the patient at home						
Carers' Alert	1 - Do you	4 - …to	2 - …with any	7 - …about		9 - …to balance	4 - …to	

Thermometer (CAT)	currently have any needs or concerns about providing care or your own health and wellbeing?<br>3 - …with any information, support, or equipment to provide personal care or general daily care?<br>11 - How able do you feel you can continue providing care at the current level for the person with MND?	provide any emotional or spiritual care the person may need?<br>10 - …to manage any feelings or worries that you may have?	information about the person's condition and how their care needs might change over time?<br>5 - …to know who to call in an emergency, or out-of-hours, to discuss any concerns about the person?<br>6 - …to feel involved in the decision-making and listened to by professionals about the care needed by the person?	financial, legal or work issues?<br>8 - …to take a break from caring during the day or overnight? (e.g. respite)<br>End-of-life question - Do you know the person's wishes and preferences for end-of-life care…?		your own needs with the demands of caring?	provide any emotional or spiritual care the person may need? (spiritual component included)	
Assessment tool in the form of a questionnaire	(a) carers' health	(b) carers' feelings<br>(c) how he or she is coping	(h) skills training and information needs	(d) financial circumstances<br>(e) accommodation circumstances	(f) support systems<br>(g) family relationships			
Caregivers' Aspirations, Realities, and Expectations Tool (CARE Tool)	physical/nursing care, household work, support/supervision, coordination (29 questions)	juggling responsibilities (12 questions)	planning-crisis, planning future (6 questions)	help received, formal services (5 questions)<br><br>financial costs (6 questions)<br><br>Housing (5 questions)	relationship with care recipient, relationships with family (10 questions)			

	physical health, mental health (5 questions)							
Palliative Care Quality Network (PCQN) Caregiver Needs Assessment (and the new Family/caregiver well-being survey)	B4 – Health and Wellness:<br><br>I worry my own health has worsened due to providing care<br><br>Intimacy or sexuality (physical wellbeing)	B1 – How supported do you feel in the care you are providing? (emotional perception of support)<br>B2 – Level of distress about caregiving role during the past week<br>B4 – Spiritual concerns (meaning, mortality → emotional/spiritual)<br>B4 – Interacting with the person I care for (emotional impact of interactions)<br>B5 – Emotional feelings over the past week:<br><br>Isolation or loneliness<br><br>Worry or anxiety	B3 – Lack of information has been a problem regarding:<br><br>Health condition<br><br>Medications<br><br>Nutrition<br><br>Navigating health care<br><br>Planning for the future<br><br>Helping make healthcare decisions<br><br>Connecting with community resources	B6 – Practical:<br><br>Transportation<br><br>Housing or living arrangements<br><br>Insurance coverage<br><br>Food<br><br>Work<br><br>Financial concerns			B4 – Spiritual concerns (facing mortality, loss in faith, meaning in life)	

		Not knowing how to best provide care (emotional uncertainty)<br><br>Anger or frustration<br><br>Sadness or depression<br><br>Feeling overwhelmed or exhausted						
Carer Need Screening Tool (CNST-11)	1. My health is not good, or I often have insomnia.<br>2. I often feel like I don't have the confidence or opportunity to take care of my own physical and mental health.	1. I feel anxious, agitated and uneasy.<br><br>2. I have thoughts of self-harm or harming others.<br><br>3. In the past 12 months, major changes in my life (e.g., moving, divorce, bereavement, unemployment, retirement, serious health issues) have		1. I am financially burdened by caring for the care recipients.<br><br>2. I am unable to provide the care needed by the care recipient.	8. When I have important decisions to make, I often feel like there is no one who can help me (including family members who live with me or elsewhere, friends, and other sources of assistance).<br><br>9. Physical or verbal	I don’t have any time for myself to take a break.		

		affected my daily life and caregiving abilities.			conflicts often occur between the care recipient and me.<br><br>10 The care recipient exhibits aggressive speech and behaviour (e.g., harasses others, behaves indecently or aggressively).			
Family Needs Survey (FNS)	Family and Social Support – 1. Talking with someone in my family about concerns<br>Family and Social Support – 6. Helping our family support each other during difficult times<br>Explaining to Others – 5. Finding reading material about	Information – 1. How children grow and develop<br>Information – 2. How to play or talk with my child<br>Information – 3. How to teach my child<br>Information – 4. How to handle my child's behavior<br>Information –	Information – 1. How children grow and develop<br>Information – 2. How to play or talk with my child<br>Information – 3. How to teach my child<br>Information – 4. How to handle my child's behavior<br>Information – 5. Information	Family and Social Support – 7. Deciding who will do household chores, child care, and other family tasks<br>Family and Social Support – 8. Deciding on and doing family recreational activities (practical coordination)	Family and Social Support – 2. Having friends to talk to<br>Family and Social Support – 4. Helping my spouse accept any condition our child might have<br>Family and Social	Family and Social Support – 3. Finding more time for myself (social/leisure participation)	Child Care – 3. Getting appropriate care for my child in a church or synagogue during religious services	

	other families who have a child like mine (emotional identification, coping) Professional Support – 1. Meeting with a counselor (psychologist, social worker, psychiatrist)	5. Information about any condition or disability my child might have Information – 6. Information about services that are presently available for my child Information – 7. Information about the services my child might receive in the future Explaining to Others – 1. Explaining my child's condition to my parents or spouse’s parents Explaining to Others – 2. Explaining my child's condition to his or her siblings Explaining to Others – 3. Knowing how to respond	about any condition or disability my child might have Information – 6. Information about services that are presently available for my child Information – 7. Information about the services my child might receive in the future Explaining to Others – 1. Explaining my child's condition to my parents or spouse’s parents Explaining to Others – 2. Explaining my child's condition to his or her siblings Explaining to Others – 3. Knowing how to respond when friends,	Financial – 1. Paying for expenses such as food, housing, medical care, clothing, or transportation Financial – 2. Getting any special equipment my child needs Financial – 3. Paying for therapy, day care, or other services Financial – 4. Counseling or help in getting a job Financial – 5. Paying for babysitting or respite care Financial – 6. Paying for toys that my child needs Child Care – 1. Locating babysitters or respite care providers Child Care – 2. Locating a day care program or preschool	Support – 5. Helping our family discuss problems and reach solutions			

		when friends, neighbors, or strangers ask questions about my child Explaining to Others – 4. Explaining my child's condition to other children	neighbors, or strangers ask questions about my child Explaining to Others – 4. Explaining my child's condition to other children	Child Care – 3. Getting appropriate care during religious services Community Services – 1. Meeting & talking with other parents (peer support but tied to navigating services) Community Services – 2. Locating a doctor who understands my child's needs Community Services – 3. Locating a dentist willing to see my child				
Care Partner Hospital Assessment Tool (CHAT)	Looking after own health; physical caregiving capacity	Managing stress; emotional strain; feeling prepared for caregiving role	Understanding care partner responsibilities; information/training needs for post-discharge care; discharge preparation; decision-making preferences	Skills training for medical/nursing tasks; discharge planning needs; equipment; medication management; care coordination	Communication with healthcare team; involvement in care discussions; inclusion in planning	Preparedness for caregiving role after discharge; balancing caregiving responsibilities		Not explicitly assessed as a standalone domain
Riffin Primary	Care recipient's health	Coping with feelings, stress,	Information about	Paying medical bills;	Speaking with	Not directly assessed	Not assessed	

Care Tool Caregiver , Healthcare Engagement Checklist (CHEC) –/ Visit Companion Checklist	conditions; medications; performing health management tasks	worries; need for respite	conditions, treatment plans, medications, next steps	insurance/benefits; arranging services (home health, meals, transportation); making appointments; navigating providers	healthcare providers; communication support (e.g., bilingual services)			
