# Supplementary material for: Mapping Caregiver Needs’ Assessment Tools for Family and Friend Caregivers: A Rapid Scoping Review
Source: Int J Environ Res Public Health. 2026 Feb 28;23(3):300. doi: 10.3390/ijerph23030300 (PMC13027153; doi:10.3390/ijerph23030300)
Supplement: Supplementary file 1 [file ijerph-23-00300-s001.zip › PRISMA Checklist.pdf]

PRISMA-ScR Checklist with Line Numbers (Completed)

TITLE

Item	PRISMA-ScR Item	Reported (Line #)
1	Identify the report as a scoping review	**Lines 2–3** (Title: *“A Rapid Scoping Review”*)

ABSTRACT

Item	PRISMA-ScR Item	Reported (Line #)
2	Structured summary (background, objectives, eligibility, sources, charting, results, conclusions)	**Lines 37–66**

INTRODUCTION

Item	PRISMA-ScR Item	Reported (Line #)
3	Rationale	**Lines 69–138**
4	Objectives	**Lines 141–151**

METHODS

Item	PRISMA-ScR Item	Reported (Line #)
5	Protocol and registration	**Lines 166–169**
6	Eligibility criteria	**Lines 183–202**
7	Information sources	**Lines 170–182**
8	Search strategy	**Lines 170–182** (full strategies in Appendix A)
9	Selection of sources of evidence	**Lines 203–213**
10	Data charting process	**Lines 217–224**
11	Data items	**Lines 218–223**
12	Critical appraisal of sources (if done)	**Not conducted;** (see **Lines 155–158, 166–169**)
13	Synthesis of results	**Lines 225–242**

RESULTS

Item	PRISMA-ScR Item	Reported (Line #)
14	Selection of sources of evidence (flow diagram)	**Lines 243–253; Figure 1 (Lines 214–215)**
15	Characteristics of sources of evidence	**Lines 254–268; Table 1 (Line 284 onward)**
16	Critical appraisal within sources (if done)	**Not applicable**
17	Results of individual sources	**Lines 273–283; Tables 1–3**
18	Synthesis of results	**Lines 290–333**

DISCUSSION

Item	PRISMA-ScR Item	Reported (Line #)
19	Summary of evidence	**Lines 334–397**
20	Limitations	**Implicit across Lines 398–411**
21	Conclusions	**Lines 466–497**

FUNDING

Item	PRISMA-ScR Item	Reported (Line #)
22	Funding	**Lines 507, 512–514**
